# Identifying gaps in gastroenterology advanced practice provider onboarding: results from a national survey and early outcomes from a pilot inpatient training curriculum

**DOI:** 10.1016/j.igie.2026.05.013

**Published:** 2026-06-02

**Authors:** Catherine Brady

**Affiliations:** Division of Gastroenterology and Hepatology, University of Colorado School of Medicine, Aurora, Colorado, USA

## Abstract

**Background and Aims:**

Advanced practice providers (APPs) play an essential role in gastroenterology (GE), yet structured onboarding practices remain highly variable across practice settings. Limited specialty-specific training may affect provider confidence, clinical readiness, and patient care quality. This study aimed to characterize national GE APP onboarding experiences and evaluate the early outcomes of a pilot inpatient GE training curriculum designed to address identified gaps.

**Methods:**

A 13-item national electronic survey was distributed through the American Gastroenterological Association (AGA) APP listserv to assess onboarding duration, confidence in managing routine GE cases, and desired training topics. Descriptive statistics, the Wilcoxon signed-rank test and McNemar test were used to analyze survey responses. On the basis of key findings, a 4-week competency-based inpatient GE curriculum was implemented at the University of Colorado (Aurora, Colorado, USA). Knowledge acquisition was measured through pretest and post-test assessments, and attending physicians evaluated APP readiness using Accreditation Council for Graduate Medical Education–aligned competency domains.

**Results:**

The survey was distributed to 289 APPs via the AGA APP listserv, with 69 responses received (67 complete; 23% response rate). Before any onboarding, 46.9% reported “not at all” or “slightly confident.” After onboarding (which was <3 months for >80% of respondents), only 39.4% felt “very” or “extremely confident” in managing routine GE cases. In the pilot curriculum, 4 APP participants with prior outpatient GE experience improved their mean knowledge scores from 9.5 out of 13 to 12 out of 13. All participants reported increased confidence, improved team integration, and greater readiness for inpatient care. Attending evaluations reflected progression from developing competence in week 1 to meeting or exceeding expectations by week 4.

**Conclusions:**

National data demonstrate substantial variability and gaps in GE APP onboarding, underscoring the need for structured, competency-based education. The pilot inpatient curriculum significantly improved knowledge, confidence, and observed clinical readiness, suggesting that short, focused training may be an effective model to enhance APP integration and support high-quality GE care.

## Background

Advanced practice providers (APPs), including nurse practitioners and physician assistants, have become essential contributors to gastroenterology (GE) care delivery, supporting the specialty's growing clinical and procedural demands.^1^ Although APPs now support a substantial portion of GE clinical services, the approach to their onboarding and training remains inconsistent among institutions.^2^^,^^3^ This study characterized onboarding practices, confidence in managing routine GE cases, and identified areas for enhanced training. As a direct response to these gaps, a 4-week inpatient GE curriculum was developed at the University of Colorado (Aurora, Colorado, USA) to strengthen knowledge and autonomy.

## Methods

A 13-item electronic survey through Research Electronic Data Capture (REDCap; Vanderbilt University Medical Center, Nashville, Tenn, USA) was disseminated via the American Gastroenterological Association (AGA) APP listserv over a 6-week period with 2 reminder emails sent every 2 weeks. Demographic data, including gender and years of experience, were not collected in the survey. Key measures included onboarding duration, confidence in managing GE cases, and desired training topics. Descriptive statistics summarized survey responses. Preonboarding and postonboarding confidence levels were collected retrospectively and compared using the Wilcoxon signed-rank test. McNemar test was used to compare proportions within individual confidence categories. Survey questions are listed in Table 1.

**Table 1:** Survey instrument: questions, response types, and response options

Item no.	Survey question	Response type	Response option
1	Are you currently, or have you previously been, used as a gastroenterology APP?	Dichotomous	Yes; no
2	How long was your onboarding training period as a GE APP? (excludes credentialing and HR onboarding)	Multiple choice	<1; 1-3; 4-6; >6 months
3	How many formal training sessions (eg, lectures, workshops, or case-based learning) were part of your onboarding?	Multiple choice	0; 1-3; 4-6; 7-10; more than 10
4	How comprehensive did you feel your training was for managing routine GE cases?	5-point Likert scale	Not at all comprehensive; somewhat comprehensive; moderately comprehensive; very comprehensive; extremely comprehensive
5	How confident were you in managing routine GE cases before your onboarding/training?	5-point Likert scale	Not at all confident; slightly confident; moderately confident; very confident; extremely confident
6	How confident were you in managing routine GE cases after completing your onboarding/training?	5-point Likert scale	Not at all confident; slightly confident; moderately confident; very confident; extremely confident
7	Do you feel your training was sufficient for your role as an independently practicing GE APP?	Dichotomous	Yes; no
8	Would you have benefited from additional formal training during onboarding?	Dichotomous	Yes; no
9	Do you believe an APP fellowship program in GE would have better prepared you for your role?	Dichotomous	Yes; no
10	If yes, what is the ideal duration for an APP GE fellowship?	Multiple choice (conditional)	3; 6; 12; 18 months; I would not benefit
11	On a scale of 0-10, how satisfied were you with your overall onboarding experience?	Numeric scale	0 (extremely unsatisfied) to 10 (very satisfied)
12	Would you recommend a fellowship curriculum for newly hired GE APPs?	Dichotomous	Yes; no
13	Please select the top 4 gastrointestinal conditions you wish you had more training on	Select up to 4	GERD; celiac disease; IBD; liver disease; CRC screening; IBS; EOE; chronic pain; acute gastrointestinal bleeding; feeding tubes and TPN

The 13-item electronic survey was developed using Research Electronic Data Capture (REDCap; Vanderbilt University Medical Center, Nashville, Tenn, USA) and distributed via the American Gastroenterological Association APP listserv over a 6-week period with 2 reminder emails. Items 4 to 6 used a 5-point Likert scale to assess training comprehensiveness and confidence before and after onboarding. Item 11 used a numeric scale (0-10) to assess overall satisfaction. Item 10 was conditional, appearing only if respondents answered “yes” to item 9. Item 13 allowed respondents to select up to 4 gastrointestinal conditions from a predefined list of 10 clinical topics. *APP,* Advanced practice provider; *CRC,* colorectal cancer; *EOE,* eosinophilic esophagitis; *GE,* gastroenterology; *GERD,* gastroesophageal reflux disease; *HR,* human resources; *IBD,* inflammatory bowel disease; *IBS,* irritable bowel syndrome; *TPN,* total parenteral nutrition.

The 4-week inpatient GE curriculum was designed using principles of competency-based medical education, including deliberate practice, progressive autonomy, and feedback-driven assessment.^4^^,^^5^ Survey-identified gaps directly informed curriculum design (Table 2). The 4-week protected training period addressed variability in onboarding duration by providing concentrated, competency-based instruction. Targeted didactics focused on areas of lowest reported confidence, and a structured evaluation framework with increasing autonomy was designed to support the transition to independent practice. Educational components included didactics from the American College of Gastroenterology Education Universe portal, case-based learning sessions, and progressive independence in clinical decision-making. Autonomy gradually expanded each week, with APPs transitioning from supervised patient care in week 1 to independent practice by week 4.

**Table 2:** Identified gaps in GE APP onboarding and corresponding curriculum design elements

Gap identified	Survey finding	Curriculum response
Onboarding duration	>80% received 3 months or less; 57% had 0 formal training sessions	Structured 4-week curriculum with protected learning time
Clinical knowledge gaps	Lowest confidence in liver disease (67%), IBD (64%), IBS/DGBI (31%), gastrointestinal bleeding (18%)	Targeted didactics on high-acuity gastrointestinal conditions
Transition to independence	39.4% low confidence postonboarding; 58% felt training insufficient	Competency-based progression with attending evaluation at weeks 1 and 4

Data derived from national survey of 67 GE advanced practice providers (APPs) distributed via the American Gastroenterological Association APP listserv. The 4 gap categories identified directly informed the design of the 4-week pilot inpatient curriculum, ensuring alignment between reported training deficiencies and educational intervention.

*DGBI*, Disorders of brain-gut interaction; *GE*, gastroenterology; *IBD*, inflammatory bowel disease; *IBS*, irritable bowel syndrome.

Metrics were collected via REDCap surveys, which were distributed to APPs and physician attendings through an e-mail link. All 4 APP participants completed identical 13-item knowledge assessments at the beginning of week 1 (pretest) and at the end of week 4 (post-test). Pretest and post-test knowledge scores were compared using paired *t* tests, with statistical significance defined as *P* < .05. Attending physicians evaluated APP clinical readiness during weeks 1 and 4 using Accreditation Council for Graduate Medical Education–aligned competency domains (Table 3). Each APP was evaluated by a single attending physician who supervised them during their assigned service week. APPs also completed an end-of-curriculum evaluation using a Likert scale to assess satisfaction and provide qualitative attitudinal feedback.

**Table 3:** Attending physician evaluation of advanced practice provider competency

Competency domain	Description	References
Patient care	Ability to provide compassionate, appropriate, and effective patient care	ACGME Core Competencies
Medical knowledge	Application of established and evolving biomedical, clinical, and cognate sciences	ACGME Core Competencies
Communication and teamwork	Interpersonal and communication skills for effective information exchange with patients, families, and health professionals	ACGME Core Competencies
Professionalism	Commitment to professional responsibilities, adherence to ethical principles, and sensitivity to diverse patient populations	ACGME Core Competencies
Systems-based practice	Awareness of and responsiveness to the larger context of health care, ability to call on system resources to provide optimal care	ACGME Core Competencies

Attending physicians evaluated advanced practice provider competency at weeks 1 and 4 using ACGME-aligned domains. Each domain was rated on a 5-point scale (1 = needs significant improvement, 2 = needs some improvement, 3 = progressing, 4 = competent, 5 = exceeds expectations). At week 4, attendings also completed a readiness assessment: “Would you recommend this fellow for independent inpatient gastroenterology work with indirect supervision?” (yes/no/with reservations).

*ACGME*, Accreditation Council for Graduate Medical Education.

## Results

### National survey outcomes

The survey was distributed to 289 APPs via the AGA APP listserv, with 67 complete responses received (23% response rate).

Three primary gaps in GE APP onboarding were identified from the survey data (Tables 4 and 5) and are summarized in Table 2 with corresponding implications for curriculum design. First, there is insufficient onboarding duration. Most respondents (>80%) reported onboarding periods of <3 months, with 24% receiving <1 month of structured training. In addition, 57% of respondents reported receiving 0 formal training sessions (eg, lectures, workshops, or case-based learning) during onboarding. Next, there is low confidence in clinical domains. Respondents identified specific gastrointestinal conditions where they felt least prepared for postonboarding. The most frequently cited training gaps included liver disease (67%), inflammatory bowel disease (64%), acute gastrointestinal bleeding (18%), disorders of brain-gut interaction/irritable bowel syndrome (31%), chronic pain management (31%), and feeding tubes/total parenteral nutrition (24%). Respondents could select multiple options. Finally, persistent confidence gaps were noted at the transition to independent practice (Fig. 1). Despite improvement over time, survey results (Tables 4 and 5) indicate that 39.4% of APPs reported less than moderately confident when beginning autonomous practice, and 58% reported that their training was insufficient for independent practice.

**Table 4:** Onboarding structure and confidence outcomes (n = 67)

Variable	n (%)
Onboarding duration <3 months	54 (80.6)
No formal training sessions (0)	38 (56.7)
Training “not at all” or “somewhat” comprehensive	30 (44.8)
Low confidence before onboarding (not at all/slightly)	58 (86.6)
Low confidence after onboarding (not at all/slightly)	26 (39.4)
Training insufficient for independent practice	37 (55.2)
Would benefit from additional training	64 (95.5)
Overall satisfaction, mean (±SD)	5.3 (±2.9)

*SD*, Standard deviation.

**Table 5:** Fellowship perspectives and training gaps (n = 67)

Variable	Category	n (%)
APP fellowship would have better prepared	Yes	58 (86.6)
Preferred fellowship duration	6 months	22 (37.9)
	12 months	22 (37.9)
Recommend fellowship for new GE APPs	Yes	61 (91.0)
Top 5 training gaps identified	Liver disease	45 (67.2)
	IBD	43 (64.2)
	Chronic pain/DGBI	22 (32.8)
	Feeding tubes/TPN	18 (26.9)
	IBS	18 (26.9)

Table 4 and this table summarize national survey findings from 67 GE APPs regarding onboarding experiences and training needs. Key findings include (1) short onboarding duration (<3 months in 80.6%), (2) limited formal training sessions (56.7% received none), (3) persistent confidence gaps despite onboarding (39.4% remained less than moderately confident), (4) strong support for APP fellowship programs (86.6% believed it would have better prepared them; 91.0% would recommend for new hires), and (5) specific clinical training gaps in liver disease, IBD, and disorders of gut-brain interaction.

*APP*, Advanced practice provider; *DGBI*, disorders of brain-gut interaction; *GE*, gastroenterology; *IBD*, inflammatory bowel disease; *IBS*, irritable bowel syndrome; *TPN*, total parenteral nutrition.

**Figure 1:**
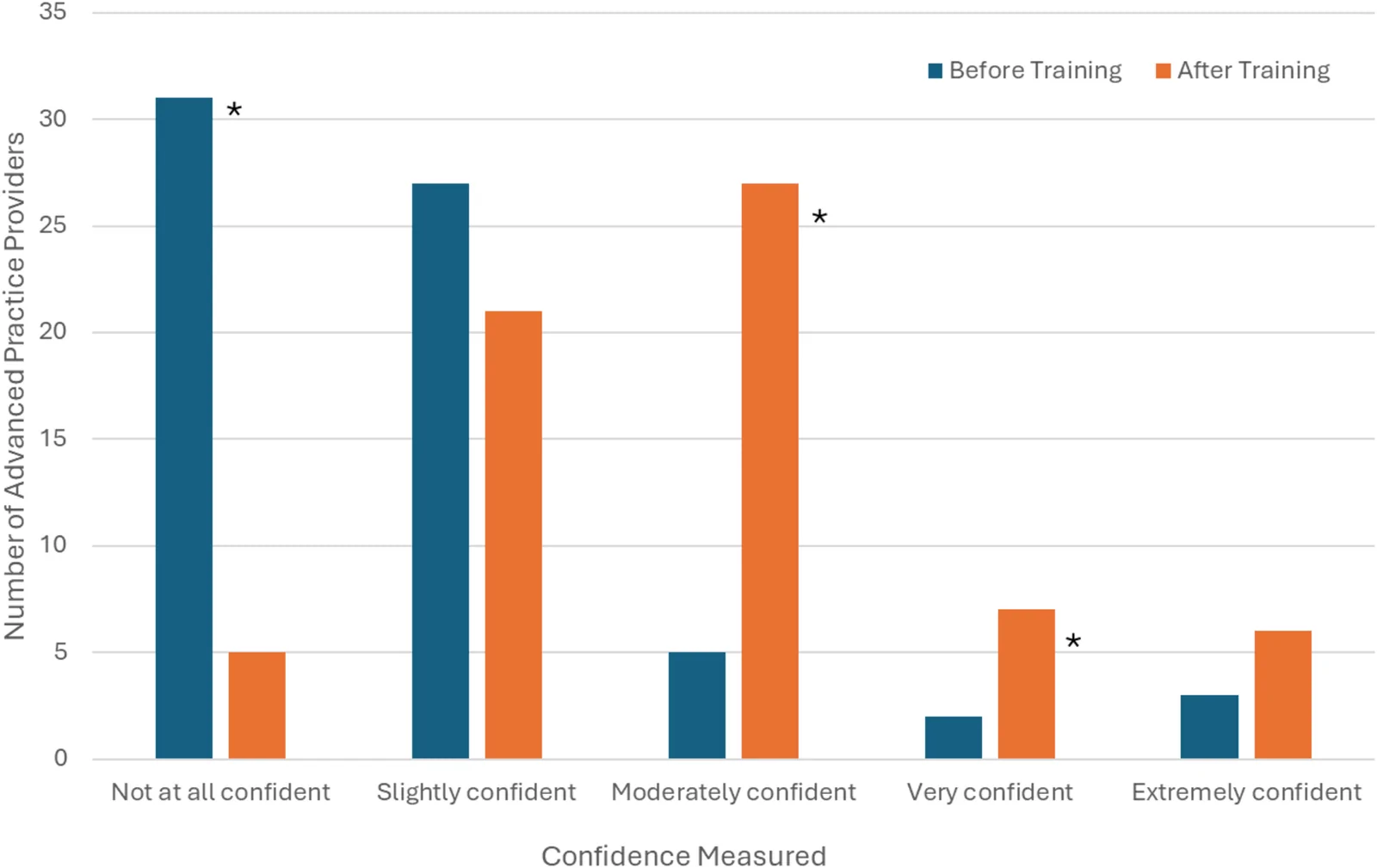
Advanced practice provider (APP) confidence in managing routine gastroenterology (GE) cases before and after onboarding: persistent gaps despite onboarding (n = 67). APP confidence in managing routine GE cases before and after onboarding (n = 67). Confidence was assessed using a 5-point Likert scale. The proportion of APPs reporting low confidence (not at all/slightly confident) decreased from 86.6% before onboarding to 39.4% after onboarding. *Asterisks* indicate statistically significant changes: “not at all confident” decreased from 46.3% to 7.6% (*P* < .001); “moderately confident” increased from 9.0% to 40.9% (*P* < .001). Despite improvement, 39.4% remained less than moderately confident at transition to independent practice.

### Pilot inpatient curriculum outcomes

Four APPs completed the 4-week inpatient GE curriculum. Pretest scores ranged from 9 to 10 out of 13 (mean 9.5 ± 0.58 standard deviation), improving to 11 to 13 out of 13 (mean 12.0 ± 0.82 standard deviation) on post-test, representing a 26% improvement in clinical knowledge (*P* < .05 by paired *t* test). Participants reported increased confidence, team integration, and clinical readiness on the curriculum evaluation. Among the APPs, 3 of 4 reported that online modules were the most helpful in learning, whereas all 4 APPs (4/4) reported that bedside teaching and case-based learning were highly effective. In addition, 2 of 4 APPs indicated that the pretests and post-tests helped guide their self-directed practice. Attending evaluations (Table 3) demonstrated APP readiness and improvement from week 1 to 4. At week 1, all 4 APPs were rated as “developing competence.” By week 4, all 4 APPs were rated as “exceeding expected performance,” demonstrating measurable advancement in clinical readiness.

## Discussion

This national survey revealed substantial variability in GE APP onboarding and persistent confidence gaps. Most respondents (80.6%) reported <3 months of onboarding, and 39.4% remained “not at all” or “slightly confident” after training. These findings highlight an urgent opportunity to design structured, competency-based onboarding that reflects the realities of GE practice.^1^^,^^2^

The pilot inpatient curriculum demonstrated measurable improvements in APP knowledge and confidence, suggesting that short, focused interventions can meaningfully enhance clinical preparedness. From a practice implementation standpoint, the pilot curriculum yielded several objective metrics that may inform decisions regarding investment in structured APP onboarding. This aligns with prior studies demonstrating the impact of structured training on provider competence.^6^^,^^7^ Improvements in mean knowledge assessment scores and progression in attending physician competency ratings represent early feasibility data from a small cohort rather than definitive return-on-investment outcomes. Practices considering investment in structured onboarding models may find these objective benchmarks useful for program justification; however, larger-scale studies are needed to confirm their effectiveness and long-term value. Notably, participants were experienced outpatient GE APPs, allowing the 4-week curriculum to build effectively on existing skills.

Several limitations warrant consideration when interpreting these findings. First, the national survey achieved a 23% response rate (67/289), introducing potential response bias, because respondents may have had stronger opinions, either positive or negative, regarding their onboarding experiences. In addition, the survey did not collect demographic variables such as gender, practice type, years since completing APP training, prior non-GE clinical experience, or whether prior experience was in inpatient versus outpatient settings.

Second, the curriculum evaluation relied primarily on knowledge assessment and self-reported confidence, which may not fully capture clinical competence or patient outcomes. Although attending evaluations provided objective assessment of readiness, longer-term follow-up examining clinical performance metrics, patient safety outcomes, and retention of knowledge would strengthen conclusions about curriculum effectiveness. In addition, the pilot curriculum used a single attending evaluator per APP at each time point (weeks 1 and 4), which may introduce evaluator bias and limits assessment of inter-rater reliability.

Lastly, although 39.4% of APPs remained less than moderately confident after completing onboarding, the survey did not include open-ended qualitative questions to determine the specific causes of this persistent low confidence. Future qualitative research incorporating semistructured interviews or focus groups is needed to better characterize these barriers and inform targeted interventions.

Despite these limitations, the consistency between national survey findings and pilot outcomes suggests that structured GE APP training addresses a genuine educational need. Broader adoption of standardized training or fellowship models could enhance workforce preparedness and improve patient care quality.^1^, ^2^, ^3^ Future research should prioritize multisite implementation studies with standardized outcome measures to establish best practices for GE APP onboarding.

## Patient consent

This article does not discuss individual patients, so no consent was needed.

## Institutional Review Board approval

Exemption was granted April 2025 (Colorado Multiple Institutional Review Board number 25-0514).

## Declaration of generative artificial intelligence and artificial intelligence—assisted technologies in the writing process

During the preparation of this work, the author used ChatGPT (OpenAI, San Francisco, Calif, USA) and OpenEvidence, Inc (Miami, Fla, USA) to assist in the writing process to improve readability and language. After using this tool/service, the author reviewed and edited the content as needed and took full responsibility for the content of the publication.

## Disclosure

The author disclosed no financial relationships.
